# Regulatory T-Cells as an Emerging Barrier to Immune Checkpoint Inhibition in Lung Cancer

**DOI:** 10.3389/fonc.2021.684098

**Published:** 2021-06-01

**Authors:** Daniel R. Principe, Lauren Chiec, Nisha A. Mohindra, Hidayatullah G. Munshi

**Affiliations:** ^1^ Medical Scientist Training Program, University of Illinois College of Medicine, Chicago, IL, United States; ^2^ Department of Surgery, Division of Surgical Oncology, University of Illinois at Chicago, Chicago, IL, United States; ^3^ Department of Medicine, Feinberg School of Medicine, Northwestern University, Chicago, IL, United States; ^4^ Robert H. Lurie Comprehensive Cancer Center, Chicago, IL, United States; ^5^ Jesse Brown VA Medical Center, Chicago, IL, United States

**Keywords:** lung cancer, immunotherapy, regulatory T (Treg) cell, Immune check inhibitor (ICI), tumor immunology

## Abstract

Immune checkpoint inhibitors (ICIs) have revolutionized the treatment paradigm for lung cancer in recent years. These strategies consist of neutralizing antibodies against negative regulators of immune function, most notably cytotoxic T-lymphocyte-associated protein 4 (CTLA-4), programmed cell death protein 1 (PD-1), and PD-1 ligand 1 (PD-L1), thereby impeding the ability of tumor cells to escape immune surveillance. Though ICIs have proven a significant advance in lung cancer therapy, overall survival rates remain low, and lung cancer continues to be the leading cause of cancer-related death in the United States. It is therefore imperative to better understand the barriers to the efficacy of ICIs, particularly additional mechanisms of immunosuppression within the lung cancer microenvironment. Recent evidence suggests that regulatory T-lymphocytes (Tregs) serve as a central mediator of immune function in lung cancer, suppressing sterilizing immunity and contributing to the clinical failure of ICIs. Here, we provide a comprehensive summary of the roles of Tregs in lung cancer pathobiology and therapy, as well as the potential means through which these immunosuppressive mechanisms can be overcome.

## Introduction

Non-small cell lung cancer (NSCLC) is the most common form of lung cancer, representing roughly 85% of all new lung cancer diagnoses (1). Most patients are diagnosed with advanced disease, stemming from inadequate screening and the late onset of symptoms (1). The treatment for NSCLC is highly varied and can include a combination of surgery, chemotherapy, radiation, targeted therapy, and most recently, immunotherapy (2). Lung cancer immunotherapy is backboned by immune checkpoint inhibitors (ICIs), which have demonstrated significant antitumor activity in most solid tumors (3–9). ICI-based immunotherapy consists of neutralizing antibodies against negative regulators of immune function, such as cytotoxic T-lymphocyte-associated protein 4 (CTLA-4), programmed cell death protein 1 (PD-1), and PD-1 ligand 1 (PD-L1), thereby limiting the ability of tumor cells to escape the cytotoxic immune program (10). However, despite the advent of immunotherapy, NSCLC carries a combined 5-year survival rate of only 25% (11). Similarly, ICI-based immunotherapy is approved as a first-line treatment for small cell lung cancer (SCLC) (12). However, patients with SCLC display even poorer outcomes, with an overall 5-year survival rate of 7% (11). Hence, there are still several remaining obstacles limiting the therapeutic efficacy of ICIs in lung cancer that must be overcome in order to further enhance drug responses and improve patient outcomes.

Recent evidence suggests that several resident immune cell subsets within the NSCLC tumor microenvironment (TME) may contribute to immune evasion, thereby blunting the effects of ICI-based immunotherapy. This includes regulatory T-lymphocytes (Tregs), a specialized T-cell subpopulation that acts to suppress sterilizing immune responses, thus promoting self-tolerance (13). While Tregs have central roles in maintaining normal airway tolerance (14), Tregs are abundant in NSCLC and predict for an increased risk of disease recurrence in early-stage disease (15). Similarly, increased tumor-infiltrating Tregs are associated with poor overall survival in SCLC (16). Here, we discuss the mechanistic contributions of Tregs to lung cancer pathobiology, as well the means through which Tregs limit therapeutic responses to ICI-based immunotherapy regimens and the means through which this can be overcome.

## Treg Differentiation and Normal Airway Tolerance

**Figure 1:**
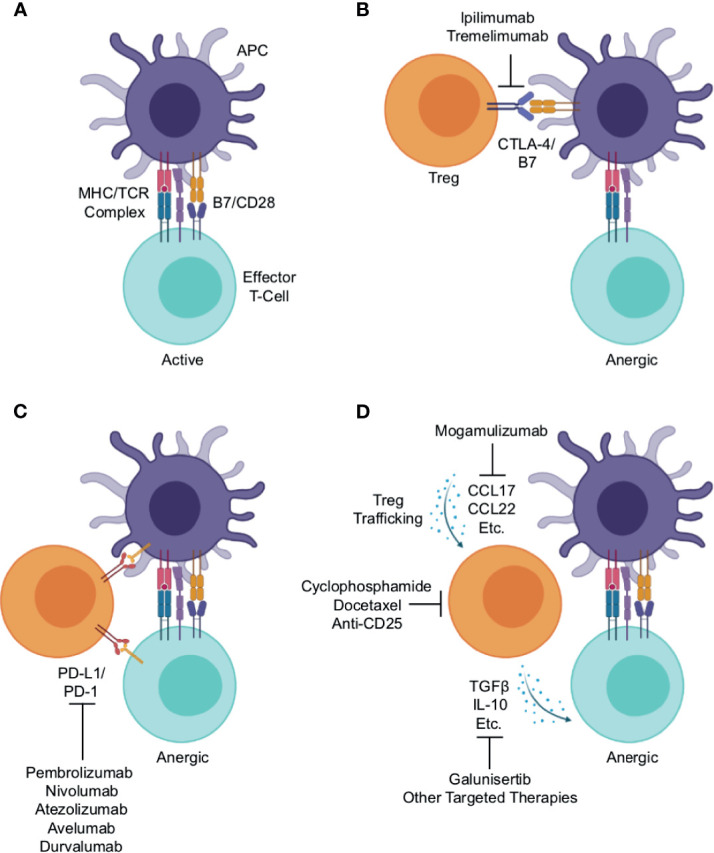
Mechanisms of Treg-mediated immune evasion within the lung cancer immune microenvironment and strategies for therapeutic intervention. **(A)** Under physiologic conditions, an activated antigen-presenting cell (APC) will associate with an effector T-cell, presenting antigen peptide on an MHC molecule. This will associate with the T-cell receptor, which combined with additional stimuli such as co-stimulation mediated in part by association of the APC’s B7 and T-cell’s CD28, lead to T-cell activation and enhanced effector function. Regulatory T-cells (Tregs) suppress effector T-cell activation through a variety of mechanisms. **(B)** One such mechanism is the association of Treg’s cytotoxic T-lymphocyte-associated protein 4 (CTLA-4) with the APC’s B7, outcompeting the effector T-cell for the co-stimulation signal thereby leading to anergy. This serves as the rationale for the use of anti-CTLA-4 antibodies such as ipilimumab and tremelimumab, which function to block this interaction, thereby enhancing anti-tumor immune responses. **(C)** Both APCs and effector T-cells can express programmed cell death protein 1 (PD-1) on their surface. Treg expressed PD-1 ligand 1 (PD-L1) can associate with PD-1, leading to reduced co-stimulation by the APC or functional inactivation of the effector T-cell. This association is interrupted by antibodies against PD-1 (pembrolizumab and nivolumab) or PD-L1 (e.g., atezolizumab, avelumab, and durvalumab), and this strategy is now considered the cornerstone of lung cancer therapy. **(D)** Tregs traffic into tumor tissues largely by following gradients of CCR4 ligands such as CCL17 and CCL22. There, they produce a variety of suppressive cytokines, namely interleukin 10 (IL-10) and transforming growth factor β (TGFβ). These both can limit effector T-cell responses, and facilitate tumor escape from immune surveillance. This also provides several potential opportunities for therapeutic intervention including: anti-CCR4 antibodies such as mogamulizumab (KW-0761) to block Treg trafficking, anti-CD25 antibodies or chemotherapy agents, cyclophosphamide and docetaxel, to deplete Tregs, or anti-TGFβ agents such as galunisertib or other targeted therapies to block the immune suppressive actions of Treg-derived cytokines within the lung tumor microenvironment.

Tregs are a subset of CD4+ T-cells, typically defined by the expression of the transcription factor Forkhead box protein P3 (FoxP3) (17). In contrast to other CD4+ T-cells that enhance local immune function, Tregs maintain immune homeostasis and self-tolerance by suppressing the activity of other immune cell subsets, thereby restraining autoimmune responses in the periphery (18–21). Treg-mediated immune suppression occurs through a variety of mechanisms. Under physiologic conditions, an activated antigen-presenting cell (APC) will display a processed antigen peptide on its surface *via* an MHC molecule. This antigen/MHC complex will then associate with the T-cell receptor (TCR) of a nearby T-cell. This interaction, when combined with an additional co-stimulatory signal mediated in part by association of the APC’s B7 and T-cell’s CD28, lead to the activation of the T-cell, which then can clonally expand and/or exert its enhanced effector function (**Figure 1A**) (22).

This provides several avenues through which Tregs can suppress the activation of a neighboring T-cell. For instance, Tregs constitutively express cytotoxic T-lymphocyte-associated protein 4 (CTLA-4) on their surface, which binds to the B7 on the APC surface, outcompeting CD28 expressed on a nearby T-cell. In the absence of a B7/CD28 interaction, the effector T-cell will remain refractory from full activation (**Figure 1B**) (23). This serves as the rationale for using anti-CTLA-4 antibodies such as ipilimumab and tremelimumab that block this interaction as a means of cancer immunotherapy, aiming to enhance anti-tumor immune responses (**Figure 1B**) (24). Similarly, both APCs and T-cells can express programmed cell death protein 1 (PD-1) on their surface. Treg expressed PD-1 ligand 1 (PD-L1) can associate with PD-1, leading to reduced co-stimulation by the APC or direct inactivation of an effector T-cell (**Figure 1C**) (23). This, in part, is the rationale for using blocking anti-PD-1 antibodies (e.g., pembrolizumab and nivolumab) or anti-PD-L1 antibodies (e.g., atezolizumab, avelumab, and durvalumab) as cancer therapy (**Figure 1C**) (25).

Finally, Tregs traffic to tissues predominantly by following gradients of CCR4 ligands such as CCL17 and CCL22 (26). At the tumor site, Tregs produce a variety of suppressive cytokines such as 10 (IL-10) and transforming growth factor β (TGFβ), which can bind receptors on other T-cells or other nearby immune cells and prevent their full activation (**Figure 1D**). This also provides several potential opportunities for therapeutic intervention discussed in detail in a subsequent section of this article. In brief, these include the use of anti-CCR4 antibodies such as mogamulizumab (KW-0761) to block Treg trafficking (26), anti-CD25 antibodies (27) or chemotherapy to deplete Tregs (28, 29), anti-TGFβ agents such as galunisertib (30), or other targeted therapies to block the immune suppressive actions of Treg-derived cytokines (**Figure 1D**).

Tregs can be induced early in thymocyte development during T-cell selection in the thymus (31) or generated in the periphery through the conversion of naïve CD4+ T-cells to Tregs (32). In the thymus, the primary factor guiding Treg differentiation appears to be the specificity of the T cell receptor (TCR). In brief, there is a range of TCR self-reactivity that is permissive for Treg differentiation, provided the appropriate co-stimulatory and cytokine signals are present in subsequent stages of development to induce FoxP3 expression (31, 33). These thymus-derived Tregs serve important roles in maintaining central tolerance, and deficiency of FoxP3 is associated with severe autoimmune disease, colitis, and allergies in mice and humans alike (34–37).

Contrastingly, the extrathymic genesis of peripheral Tregs involves the conversion of naïve Tregs in the periphery, and is primarily dictated by the local cytokine milieu, though still dependent on FoxP3 expression following antigen exposure (32). Though peripheral Tregs represent a small percentage of total Tregs under physiologic conditions, peripheral Tregs are strongly represented in the gastrointestinal tract and placenta, and maintain immune tolerance toward commensal bacteria, ingested antigens, allergens, and the fetus during pregnancy (37). Peripheral Tregs have been suggested to be the main Treg subset represented in most cancers, and are thought to be converted within the tumor microenvironment (38), though this warrants continued exploration. To date, most studies have focused on the contributions of transforming growth factor β (TGFβ) signaling in peripheral Treg conversion. TGFβ has been shown to induce FoxP3 expression in both human and murine T-cells, and TGFβ-induced Tregs are central to preventing house dust mite-induced allergic lung pathogenesis in a murine model of asthma (39–41). Similarly, blockade of TGFβ signaling has been shown to reduce FoxP3 expression in *ex vivo* T-cell cultures (42), as well as disrupt Treg-mediated tolerance to inhaled antigen *in vivo* (43).

These and other studies suggest that Tregs play essential roles in maintaining normal airway tolerance. For instance, a seminal study demonstrated that animals with a selective deficit in extrathymically generated Tregs spontaneously develop pronounced T-helper 2 (Th2)-associated pathologies at mucosal sites in the lungs, specifically in the form of allergic inflammation and asthma (44). These and several other studies have substantiated Tregs as a central mediator of immune responses to inhaled antigens, limiting the activation of pathogenic immune cells and preventing tissue-damaging inflammatory responses (14, 45). Importantly, these studies also suggest that Treg-dependent maintenance of airway tolerance requires continuous exposure to airborne antigens, as antigen withdrawal results in a decreased number of Tregs, enhancing susceptibility to pathologic Th2-dependent response against respiratory antigens (46, 47).

## Tregs as a Prognostic Biomarker in Lung Cancer

In addition to maintaining airway tolerance, Tregs are also an established component of the lung cancer microenvironment, functioning to inhibit autologous T-cell proliferation and impede local immune responses (48). This has led to the long-standing hypothesis that Tregs have driving roles in lung cancer pathogenesis, by inhibiting the action of auto-reactive T-cells and allowing for the continued escape from immune surveillance. As the relative abundance of Tregs has been shown to predict for poor clinical outcomes in most cancer types (49), several studies have explored Tregs as a potential prognostic biomarker in lung cancer (**Table 1**). A recent pan-cancer meta-analysis determined that lung cancer patients with higher tumor densities of FoxP3+ Tregs had significantly poorer disease-free survival rates (49). A lung cancer-specific meta-analysis similarly found that, in a combined cohort of 1,303 NSCLC patients across 11 studies, an increase in tumor-infiltrating FoxP3+ Tregs was associated with poor overall survival. Interestingly, the authors also found that the Treg infiltrate was strongly associated with smoking status, though they did not find a relationship between Tregs and other clinicopathological features (50). Similarly, a large study conducting gene expression analysis of 196 NSCLC and 137 normal samples found that immune-infiltrating Treg-related genes were strongly associated with poor overall survival (55).

**Table 1:** Studies exploring regulatory T cells as a prognostic biomarker in lung cancer.

Cancer Type(s)	Number of Patients	Treg Location	Treg Definition(s)	Method(s)	Outcome	Reference
Pan-cancer Meta Analysis	15,512 (251 NSLC)	Tumor tissue	FoxP3+ cells	IHC	Tumors with high tumor densities of FoxP3+ Tregs are associated with poorer disease-free survival	(49)
NSCLC Meta Analysis	1,303	Tumor tissue	FoxP3+ cells	IHC	Increased FoxP3+ Tregs associated with poor overall survival and smoking status	(50)
NSCLC	100 (Complete resection)	Tumor tissue	FoxP3+ cells	IHC	Increased tumor-infiltrating Tregs predicted for earlier recurrence in node-negative NSCLC	(51)
NSCLC	87	Tumor tissue	FoxP3+	IHC	Increased tumor-infiltrating Tregs was associated with poor overall and relapse-free survival	(52)
NSCLC	196	Tumor tissue	FoxP3+	IHC	Increased intratumoral Tregs predicted for poor overall survival	(53)
NSCLC	110	Tumor tissue	FoxP3+	IHC	Increased Tregs were associated with male sex, regional lymph node involvement, advanced clinical stage, and poor overall survival. Patients with the highest expression of B7-H3+FoxP3+ Tregs had the poorest survival of all groups	(54)
NSCLC	333 (196 NSCLC, 137 Normal)	Tumor tissue	N/A	Gene Expression Analysis	Tumors with increased expression of Treg-related genes were associated with poor overall survival	(55)
NSCLC (Stage I)	64	Peripheral blood	FoxP3+ cells	IHC	An increased proportion of Tregs relative to total tumor-infiltrating lymphocytes was associated with a higher risk of recurrence and worse clinical outcomes	(15)
NSLC (Stage III/IV)	156 (Chemo-naïve)	Peripheral blood	CD4+CD25high cells were sub-classified as either: Naïve (CD127−/lowCD152-FoxP3lowCD45RO−) Effector (CD127lowCD152+FoxP3+CD45RO+) or Terminal Effector (CD127−CD152+FoxP3+CD45RO+)	FC	Increased terminal effector Tregs was associated for improved overall and progression-free survival, and increased naïve or effector Tregs with worse survival	(56)
NSCLC	70 (Receiving RT)	Peripheral blood	CD4+CD25+CD127low	FC	Increased peripheral blood Tregs was associated with poor progression-free survival	(57)
NSCLC	64 (45 chemo naïve, 19 chemotherapy treated)	Peripheral blood and tumor tissue	Thymus-derived: CD4+CD25+Helios- Peripherally generated: CD4+CD25+Helios-	FC	Patients with reduced Helios expression in tumor-infiltrating Tregs had significantly poorer survival	(58)
SCLC	65	Tumor tissue	FoxP3+	IHC	Increased tumor-infiltrating Tregs was associated with poor overall survival	(16)

NSCLC, Non-small cell lung cancer; SCLC, Small cell lung cancer; Tregs, Regulatory T cells; FC, Flow Cytometry; IHC, Immunohistochemistry; RT, Radiotherapy.

Interestingly, peripheral blood Tregs increase in a stage-dependent manner in NSCLC, and have also been suggested to have potential utility as a prognostic biomarker (59). One of the earliest studies evaluating Tregs and lung cancer prognosis included 64 stage I NSCLC patients. Using excisional biopsies, the authors found that 81% of samples had detectable tumor-infiltrating lymphocytes, with 51% positive for FoxP3+ Tregs. They then determined that patients with a higher proportion of tumor Tregs relative to total tumor-infiltrating lymphocytes had a significantly higher risk of recurrence and worse clinical outcomes (15). Similarly, a recent study evaluated peripheral blood samples from 156 chemotherapy-naive patients with stage III or IV NSCLC. Similar to studies exploring tumor-infiltrating Tregs, the authors observed a significant relationship between peripheral Tregs and clinical outcomes. This study also stratified Tregs into three additional subsets based on surface marker expression: naïve, effector, and terminal effector. Naive Tregs were defined as being CD4^+^CD25^high^CD127^−/low^CD152^-^FoxP3^low^CD45RO^−^, and represent an early stage of Treg differentiation with reduced sensitivity to apoptotic stimuli (60). Effector Tregs were those that were CD4^+^CD25^high^CD127^low^CD152^+^FoxP3^+^CD45RO^+^, and represent a transient stage of Treg differentiation that rapidly divide prior to their disappearance (61). Terminal effector Tregs were defined as CD4^+^CD25^high^CD127^−^CD152^+^FoxP3^+^CD45RO^+^, and are considered the most immune-suppressive Treg subtype (62, 63).

Using this approach, they determined that the increased presence of terminal effector Tregs predicts for improved overall and progression-free survival, whereas an increase in either naïve or effector Tregs was associated with worse survival (56). Similarly, the increased presence of peripheral blood Tregs has also been suggested to predict for clinical responses to radiotherapy. In a group of 70 NSCLC patients undergoing radiation, increased Tregs independently predicted for poor progression-free survival (57). This may be particularly noteworthy given the frequent cooperation between radiation and immunotherapy in several cancers (64), and warrants continued exploration.

Tregs have also been shown to have potential utility in predicting for poor survival in NSCLC patients undergoing definitive surgery. In a retrospective analysis of 100 patients who had undergone a complete resection for NSCLC, the authors evaluated the prognostic utility of the combination of epithelial Cyclooxygenase-2 (COX-2) expression and tumor-infiltrating Tregs. In this group, patients with high COX-2 expression had significantly worse recurrence-free survival, accompanied by a relative increase in Treg infiltration. They found that only lymph node involvement was an independent predictor of recurrence-free survival in a multivariate analysis. However, in node-negative NSCLC, they determined that FoxP3+ tumor-infiltrating Tregs was an independent predictor of shorter recurrence-free survival (51).

A similar study of 196 NSCLC patients found improved overall survival for patients with increased tumor-infiltrating CD8+ T-cells, but poorer overall survival for patients with increased tumor-infiltrating FoxP3+ Tregs (53). While these studies have relied on FoxP3+ Tregs, others have included additional parameters, including FoxP3 expression in tumor cells. For example, a study of 87 excisional biopsies from operable NSCLC patients found that FoxP3+ tumor cells were found in 31% of lung cancer specimens, with no significant relationship to tumor-infiltrating Tregs. Further, increased tumor-infiltrating Tregs were associated with poor overall and relapse-free survival, and though tumor expression of FoxP3 was not an independent predictor of outcomes, when FoxP3+cancer cells were present, the relationship between tumor Treg infiltration and worse prognosis was attenuated. Conversely, patients without FoxP3- tumor cells and high Tregs had worse outcomes than all other groups (52).

In addition to tumor cell expression of FoxP3, there are other markers that may also influence the prognostic utility of Tregs. One such example is the transcription factor Helios, which has been suggested as a means of differentiating between thymus-derived and peripherally induced Tregs (65). Helios-expressing Tregs are generated during thymic selection, whereas Helios-non-expressing Tregs represent those induced in the periphery. These two Treg subsets are functionally distinct, and have largely non-overlapping TCR reservoirs (66). In a cohort of 64 NSCLC patients, 45 of whom had undergone surgery and 19 that had received only chemotherapy, Helios was expressed in 47.5 ± 13.3% in peripheral blood and 18.1 ± 13.4% of tumor-infiltrating Tregs. Patients with reduced Helios expression in tumor-infiltrating Tregs had significantly poorer survival, suggesting that peripherally induced Tregs are a more significant driver of immune evasion in the lung TME (58). As no other study to date has attempted to differentiate between thymus-derived and peripherally induced Tregs in lung cancer, this warrants continued exploration.

Additionally, the co-inhibitory signal B7-H3 may also influence the relationship between Tregs and prognosis. In a group of 110 NSCLC specimens, FoxP3 expression in tumor-infiltrating T-cells was associated with male gender, regional lymph node involvement, advanced clinical stage, and poor overall survival. B7-H3 expression was strongly associated with tumor-infiltrating FoxP3+ Tregs, and patients with the highest expression of B7-H3+FoxP3+ Tregs had the poorest survival of all groups (54).

Though several studies have affirmed the utility of Tregs as a predictor of clinical outcomes in NSCLC, Tregs are less established as a prognostic marker in small cell lung cancers (SCLC). There is emerging evidence supporting a similar role for Tregs in SCLC, namely that several SCLC tumor cell lines can induce *de novo* differentiation of Tregs from naïve peripheral blood lymphocytes in an IL-15 dependent mechanism. The same study also evaluated SCLC tumor biopsies, and found that an increase in tumor-infiltrating Tregs was associated with poor overall survival, similar to previous studies in NSCLC (16).

## Tregs as a Predictor of Therapeutic Responses to ICIs

Given the advent of ICIs in lung cancer treatment, there is considerable interest in identifying clinically useful biomarkers to predict for therapeutic responses. While PD-L1 expression is perhaps the most widely used predictive biomarker, emerging data suggests that several other factors may be as or possibly more informative than established biomarkers, e.g., PD-L1 status. For instance, several studies have now indicated that the presence of Tregs may also have utility as a predictor of responses to ICI-based immunotherapy (**Table 2**). For example, a recent study evaluated patients with NSCLC, gastric cancer, and malignant melanoma that were treated with the anti-PD-1 antibodies nivolumab or pembrolizumab, or the anti-PD-L1 antibody atezolizumab. The authors found that non-responsive patients typically displayed increased PD-1 on Tregs. The ratio of tumor-infiltrating PD-1+CD8+ T-cells relative to Tregs was a superior predictor of therapeutic responses than all other predictors, including PD-L1 expression and tumor mutational burden. The authors therefore concluded that PD-1+ Tregs might have utility as a predictive biomarker, and warrant consideration as a therapeutic target to augment the clinical efficacy of ICIs in lung cancer (67).

**Table 2:** Studies exploring regulatory T cells as a predictor of responses to immune checkpoint inhibition.

ICI	Cancer Type(s)	Number of Patients	Treg Location	Treg Definition(s) by FC	Outcome	Reference
Nivolumab, pembrolizumab, or atezolizumab	NSCLC, gastric cancer, and malignant melanoma	39 (15 NSCLC) Discovery Cohort	Tumor tissue	Naive Tregs: CD4^+^CD25^low^FoxP3^low^CD45RA^+^ Effector Tregs (eTregs): CD4^+^CD25^high^FoxP3^high^CD45RA^-^	Poor responses to ICIs was associated with increased Treg expression of PD-1, particularly for eTregs	(67)
		48 (12 NSCLC) Validation Cohort	Tumor tissue	See Above	See Above	
Nivolumab or pembrolizumab	NSCLC	73 (31 treated with ICIs)	Peripheral blood and tumor tissue		Increased frequency of tumor-infiltrating, PD-L1^high^ Tregs was associated with improved responses to PD-1 inhibition	(68)
Nivolumab or pembrolizumab	NSCLC	83 Discovery Cohort	Peripheral blood	Effector Tregs (eTregs): CD4^+^CD25^+^FoxP3^+^CD45RA^-^	Increased peripheral eTregs following anti-PD-1 administration predicts for improved therapeutic responses	(69)
		45 Validation Cohort	Peripheral blood	See Above	See Above	

ICI, Immune checkpoint inhibitor; NSCLC, Non-small cell lung cancer; SCLC, Small cell lung cancer; Tregs, Regulatory T cells; FC, Flow Cytometry.

A similar study evaluated peripheral blood samples and tumor specimens from 73 NSCLC patients, 31 of whom had received either pembrolizumab or nivolumab. Using this approach, the authors identified a population of high-PD-L1+, CD25+ Tregs (PD-L1^hi^ Tregs) that was preferentially expressed within tumor tissues and correlated with PD-1 expression in tumor-infiltrating CD8+ T-cells. Patients with an increased frequency of tumor-infiltrating PD-L1^hi^ Tregs showed high PD-1/PD-L1 pathway dependence, and improved CD8+ T-cell responses following PD-1 inhibition. This corresponded to improved clinical outcomes compared to patients with a low frequency of PD-L1^hi^ Tregs (68).

A recent study has also explored peripheral Tregs as a potential biomarker for responses to ICIs. One such study evaluated peripheral blood samples from 83 NSCLC patients before and after ICI-based immunotherapy. In this group, patients with a high frequency of Tregs one week after anti-PD-1 administration had significantly improved response rates, as well as longer progression-free and overall survival, though the number of peripheral Tregs prior to therapy had no predictive value. Similar results were observed regarding serum levels of TGFβ, which was also associated with improved clinical outcomes. These results were affirmed in a second cohort of 45 patients, suggesting that increased peripheral Tregs or elevated levels of TGFβ can also predict for clinical outcomes (69).

## Tregs as a Therapeutic Target

As discussed, given the many roles of Tregs in blunting the effector function of CD8+ T-cells, Tregs have long been suggested as a barrier to the efficacy of ICIs and a potential target for therapy (70). This approach has shown encouraging preclinical efficacy, particularly combined with other treatment modalities such as radiation (71–73). Several strategies to deplete or modulate the activity of Tregs have been introduced (**Figure 1**). Though it has been suggested that the efficacy seen with CTLA-4 inhibitors may be mediated in part through the depletion of Tregs, recent evidence suggests that CTLA-4 inhibitors do not significantly change or deplete Tregs within the tumor microenvironment (74). However, the effects of select chemotherapy agents on Tregs are well documented, particularly regarding cyclophosphamide (75).

The tumoricidal effects of cyclophosphamide appear primarily dependent on the immune system, as a single injection of high-dose cyclophosphamide increased the survival of immunocompetent mice, though this was not observed in immune-deficient mice (76). Accordingly, Tregs are highly sensitive to cyclophosphamide, particularly when compared to CTLs and helper T cells, which was presumed to be due to a DNA repair defect (77). This has been shown to be a central mediator of cyclophosphamide-induced type-1 diabetes in mice, which was prevented by the allogenic transfer of Tregs (78). Likewise, *in vitro* studies demonstrate that low-dose cyclophosphamide has been reported to induce Treg apoptosis, restrain Treg proliferation, and limit their immunosuppressive functions (79).

Thus, cyclophosphamide has been suggested as a potential means of targeting Tregs in cancer, particularly in light of observations that cyclophosphamide-mediated depletion of Tregs allows immunotherapy to be curative in a rat model of implanted PROb colon cancer cells (80). Similar results have been observed in a variety of mouse models. Cyclophosphamide reduced regulatory T-cells, enhancing the efficacy of non-myeloablative allogeneic stem cell transplantation through increased activation of autoreactive T-cells and IFN-γ production (81). In a mouse model of cutaneous melanoma, cyclophosphamide has also been demonstrated to increase the frequency of tumor-infiltrating, IFN-γ producing T-cells, as well as decrease the relative abundance of Tregs (82). In colon cancer patients, low-dose cyclophosphamide depleted tumor-associated Tregs, enhanced anti-tumor immune responses, and extended overall survival (29). Though encouraging, the clinical utility for cyclophosphamide as an adjuvant to immunotherapy is still emerging in lung cancer.

Other chemotherapy agents have also been suggested to selectively target Tregs. For example, docetaxel has been shown to selectively reduce Tregs *in vitro*, and patients who received four cycles of docetaxel-based chemotherapy showed fewer peripheral Tregs than at baseline (28). The multidrug regimens FOLFOX (5-FU, leucovorin, and oxaliplatin) and FOLFIRI (5-FU, leucovorin, and irinotecan) also significantly reduced peripheral blood Tregs in patients with metastatic colorectal cancer (83). Hence, the mechanistic intersection between chemotherapy-mediated Treg depletion and lung cancer immunotherapy warrants continued exploration (29).

Other therapeutic strategies more directly targeting Tregs are also emerging. For example, the high-affinity IL-2 receptor CD25 (also known as IL-2 receptor alpha) is strongly expressed on Tregs, and neutralizing antibodies against CD25 have been suggested as a means of Treg depletion. This approach has been highly effective in reducing Tregs in preclinical models, enhancing CD8-mediated anti-tumor immune function (27, 84). Though anti-CD25 has shown limited efficacy against established tumors, select studies have evaluated the combination of anti-CD25-mediated Treg depletion and ICI-based immunotherapy. One such study assessed the combination of anti-CD25 and anti-PD-1 in mouse models of tumorigenesis and found that this approach promoted complete tumor rejection, substantiating CD25 as a therapeutic target for combination approaches in immuno-oncology (85). Anti-CD25 has also been used in combination with near-infrared photoimmunotherapy (NIR-PIT), a method of treating cancer using the activation of an antibody-photoabsorber conjugate activated by NIR light (86, 87). Anti-CD25 guided NIR-PIT led to the selective depletion of Tregs, as well as robust CD8+ T-cell and natural killer cell activation in models where anti-CD25 alone was ineffective at depleting Tregs (87).

While these and other related findings support anti-CD25 as a potentially useful means to deplete Tregs in cancer therapy (88–90), other approaches are also showing early promise. The chemokine receptor CCR4 is expressed on 90% of Tregs and has been linked to ICI-resistance. ICIs have been suggested to upregulate CCR4 ligands, e.g., CCL17 and CCL22, thereby increasing Treg trafficking into tumors. This was ameliorated through the addition of a pharmacological inhibitor of CCR4, substantiating CCR4 as a potential means of targeting Tregs for cancer therapy (91). CCR4-inhibition has also been suggested as a means of depleting Tregs for cancer immunotherapy, augmenting cytotoxic T-cell responses (92). This approach has been explored in clinical trials, where the anti-CCR4 antibody mogamulizumab has been shown to deplete Tregs and potentially enhance cytotoxicity in adult T-cell leukemia/lymphoma (26). However, though mogamulizumab successfully depleted Tregs in lung cancer patients (93), a recent multi-cancer trial determined that the combination of mogamulizumab and durvalumab or tremelimumab does not result in improved efficacy for patients with advanced solid tumors (94). Hence, the potential for mogamulizumab as an adjuvant to ICIs in lung cancer is unclear at this time and requires further investigation.

Additional strategies are targeting Treg-derived cytokines, namely TGFβ. TGFβ is a potent and pleiotropic cytokine with several, often contradictory, roles in tumorigenesis (95). TGFβ has been shown to contribute to immune evasion in cancer (96) and attenuate therapeutic responses to PD-L1 inhibition by contributing to T-cell exclusion (97). Accordingly, the combination of TGFβ signal inhibition and ICIs are showing promise in several solid tumors (30, 98–100). Early results for bintrafusp alfa (a bifunctional fusion protein of the extracellular domain of the type 2 TGFβ receptor fused to an anti-PD-L1 antibody) showed promising efficacy and manageable toxicity in NSCLC patients previously treated with platinum-based immunotherapy (101). This has been supported by additional studies, with durable immune responses observed in many NSCLC patients after two-years, particularly those with high PD-L1 expression (102).

## Perspective

The immunosuppressive effects of Tregs in lung cancer are well documented with several recent studies affirming the prognostic relevance of circulating and tumor-infiltrating Tregs alike. Emerging evidence also supports the longstanding hypothesis that Tregs have a driving role in the clinical failure of ICI-based immunotherapy. Hence, Tregs and their associated cell processes may represent a promising therapeutic target in lung cancer, particularly in the setting of ICIs. However, despite the promise of targeting Tregs in lung cancer, there are several important distinctions that must be made prior to advancing such strategies in the clinic. As highlighted in this review, Tregs are a highly heterogeneous T-cell subset with several subcategories including thymus-derived, peripherally generated (extrathymic), naïve, effector, terminal effector, and others. Though many studies support Tregs as a potential prognostic biomarker and/or predictor of treatment failure, very few such studies account for this variance, with most using the generalized term “Tregs”. Additionally, the criteria used to define these Tregs are highly varied, both regarding methodology and the surrogate markers used. Hence, the true utility of Tregs as a predictive biomarker remains unclear, and warrants continued exploration using standardized methodology, as well as particular attention to the many unique and functionally distinct Treg subsets.

Finally, it is also important to note that targeting Tregs requires extreme caution. For instance, in a murine model of pancreatic cancer, genetic ablation of Tregs led to extensive remodeling of the tumor microenvironment and rapidly accelerated tumor formation (103). Hence, the combination of Treg-targeted therapy and ICIs warrants careful study prior to advancing to clinical practice. Additionally, Tregs (particularly those developed during thymic selection) have a central role in suppressing autoreactive T-cells and restraining tissue inflammation. Therefore, it remains a distinct possibility that broadly targeting Tregs in tandem with ICI-based immunotherapy may also lead to severe autoimmune-mediated adverse effects. Hence, this too warrants additional study, as do whether unique Treg subsets can be safely targeted for therapy (e.g., peripherally-induced Tregs) while still offering a potential clinical benefit to lung cancer patients, as this may be a useful means of maximizing efficacy while also limiting toxicity.

## Summary

ICIs are the cornerstone of lung cancer therapy. Though a significant advance, overall survival rates remain poor. Hence, there is a clear need to identify new strategies to further improve the efficacy of ICI-based immunotherapy. Tregs represent a highly suppressive T-cell subset that is abundant within the lung cancer TME. Accordingly, Tregs have many roles in maintaining peripheral tolerance and impeding anti-tumor immunity in lung cancer, likely contributing to the clinical failure of ICIs. While select strategies to deplete Tregs or neutralize their immunosuppressive effects are showing early promise, these are most effective when combined with other treatment modalities. Hence, further study is required to determine the most appropriate combinations of Treg-targeted therapies and ICI-based immunotherapy in order to maximize efficacy and minimize off-target toxicity.

## Author Contributions

DP drafted the manuscript and assembled figures. LC, NM, and HM edited the manuscript. All authors contributed to the article and approved the submitted version.

## Funding

This work was supported by NIH F30CA236031 to DP and Veterans Affairs LPOP Award I50CU000179 to HM and NM.

## Conflict of Interest

The authors declare that the research was conducted in the absence of any commercial or financial relationships that could be construed as a potential conflict of interest.
